# Effect of fecal microbiota transplantation on diabetic wound healing through the IL-17A–mTOR–HIF1α signaling axis

**DOI:** 10.1128/aem.02019-24

**Published:** 2025-02-28

**Authors:** Chenmei Peng, Pan Lei, Hongying Qi, Qianjun Zhu, Chushun Huang, Ju Fu, Chengyu Zhao

**Affiliations:** 1 Qinghai University Affiliated Hospital, Qinghai University 610469 , Xining, China; 2 Department of General Practice Medicine, Qinghai University Affiliated Hospital 610469 , Xining, China; 3 Department of Endocrinology, Qinghai University Affiliated Hospital 610469 , Xining, China; 4 Department of Endocrinology, Qinghai Province People’s Hospital, Xining, China; 5 Department of Geriatrics, Qinghai University Affiliated Hospital 610469 , Xining, China; Centers for Disease Control and Prevention, Atlanta, Georgia, USA

**Keywords:** diabetic wound, fecal microbiota transplant, IL-17A, mTOR, HIF1α

## Abstract

Diabetes is the third most common chronic disorder worldwide. Diabetic wounds are a severe complication that is costly and often results in non-traumatic lower limb amputation. Recent investigations have demonstrated that the gut microbiota as a “virtual organ” can regulate metabolic diseases like diabetes. Fecal microbiota transplantation (FMT) is an innovative therapeutic approach for promoting wound healing, but its function remains incompletely defined. A diabetes model was established by supplying mice with a high-fat diet and performing an intraperitoneal injection of streptozotocin. Diabetic wounds were then created, followed by bacterial transplantation. The relevant indexes of wound healing were evaluated to verify the promoting effect of FMT on the diabetic wounds. Human skin keratinocytes were also cultured, and cell scratch experiments were conducted to further investigate the underlying mechanism. The FMT regulated the levels of specific bacteria in the diabetic mice and helped restore the balance of intestinal microbes. This transplantation also enhanced wound healing in the diabetic mice by augmenting the closure rate, accelerating re-epithelialization, and boosting collagen deposition in skin wounds. Furthermore, FMT promoted the production of IL-17A, which significantly enhanced the growth and movement of human keratinocytes. Inhibiting molecules related to the IL-17A–mTOR–HIF1α signaling axis were shown to hinder wound re-epithelialization.This study clarifies the function of the IL-17A–mTOR–HIF1α signaling axis in the utilization of FMT in diabetic wound healing, providing a new therapeutic method and target for promoting the healing of diabetic wounds.

**IMPORTANCE:**

The Intestinal microbiota, as the organ with the largest number of microorganisms in the body, plays a crucial role in the physiological functions of the human body. Normal microbiota can be involved in various functions such as energy absorption, metabolism, and immunity of the body, and microbiota imbalance is related to many diseases such as obesity and diabetes. Diabetes, as one of the world’s three major chronic diseases, is a significant health issue that troubles more than a billion people globally. Diabetic wounds are a problem that all diabetic patients must confront when undergoing surgery, and it is an important cause of non-traumatic amputations. Exploring the role of intestinal microorganisms in the wound-healing process of diabetic mice can offer the possibility of using microorganisms as a therapeutic means to intervene in clinically related diseases.

## INTRODUCTION

Diabetes mellitus (DM) is the third most common chronic disease globally. Approximately 537 million individuals globally are affected by diabetes, and this figure is expected to rise to 783 million by the year 2045 (1). Furthermore, approximately 352 million people present with impaired fasting blood sugar or impaired glucose tolerance, and 5%–10% of these people progress to type 2 diabetes mellitus (T2DM) annually (2). Diabetes complications can significantly impact patients’ quality of life and create substantial social and economic burdens (3). Diabetic wounds represent a severe and expensive complication (4) that is challenging to heal owing to factors such as infection, peripheral vascular disease, sensorimotor disease, and autonomic neuropathy (5). Diabetes is a primary contributor to non-traumatic amputations of the lower limbs and often results in severe foot ulcers, wound gangrene, and potential amputation, placing a significant psychological, physical, and economic burden on patients. Studies have shown that the gut microbiota is associated with multiple conditions such as obesity (6), type 2 diabetes (7), intestinal diseases (8), aging (9), and cancer (10). The gut microbiota is now considered a virtual organ that regulates various systems within the human organism (11). Several studies have examined the gut microbiota of diabetic individuals compared to their non-diabetic counterparts, revealing a dysbiosis in the intestinal microbiota of diabetic individuals that elevates their risk of developing T2DM (12, 13). Consequently, modulation of the intestinal microbiome in the management of T2DM and associated conditions has emerged as a significant area of contemporary research.

Fecal microbiota transplantation (FMT) is a natural approach to re-establishing the equilibrium of the gut microbiota, aiming to restore a diverse and healthy gut microbiota while assisting in the treatment of various ailments. The clinical application of FMT has become increasingly widespread and mature. In 2013, FMT was incorporated into the clinical recommendations in the United States and recommended for cases of recurrent *Clostridium difficile* infection (14). The clinical application of FMT has garnered significant attention. The largest international FMT study to date involved 8,547 patients and over 90,000 treatments. The study also had the longest follow-up time of 5 years, providing substantial medical evidence for the safety and effectiveness of the long-term clinical application of FMT (15). Moreover, there is growing evidence of significant interactions between gut microbiota and both diabetic pathology and wound healing. Diabetic wounds frequently result in severe complications owing to the intricate interplay of diabetes with skin repair processes. FMT has demonstrated significant promise in the treatment of metabolic disorders, including diabetes (16). The gut microbiota can also interact with the skin microbiota to promote normal skin healing (17). At present, however, there are few reports about the relationship between FMT and diabetic wounds.

Recent research has indicated that the bacteria and fungi present in the gut microbiota can enhance the production of IL-17A in Th17 cells (18, 19). Natural Th17 cells are found in the gut in a microbe-dependent manner (18), and they affect multiple areas including infections, tumors, and autoimmune disorders by secreting IL-17 and IL-22 to establish defense mechanisms (20). IL-17 is a key cytokine in host mucosal protection and a target for drugs that manage different autoimmune and inflammatory conditions (21). After producing IL-17A, Th17 cells act by binding IL-17RA and IL-17RC. IL-17A acts on the IL-17 receptor in epithelial cells to activate the protein kinase B (AKT) signaling route to control the mammalian target of rapamycin (mTOR) (22) and then regulate the expression of hypoxia-inducible factor 1α (HIF1α) (23). HIF1α plays a positive role in wound healing, which can promote glycolysis to provide energy for wound healing in a microhypoxic environment (24). It can also promote the formation of new blood vessels in diabetic wounds, thereby facilitating the diabetic wound healing (25). Therefore, the role of cytokines such as IL-17A in diabetic wound healing warrants further investigation.

The aim of this research is to assess the effect of FMT treatment on diabetic wound healing and to investigate the associated mechanisms.

## RESULTS

### FMT promotes wound healing in T2DM mice

**Fig 1:**
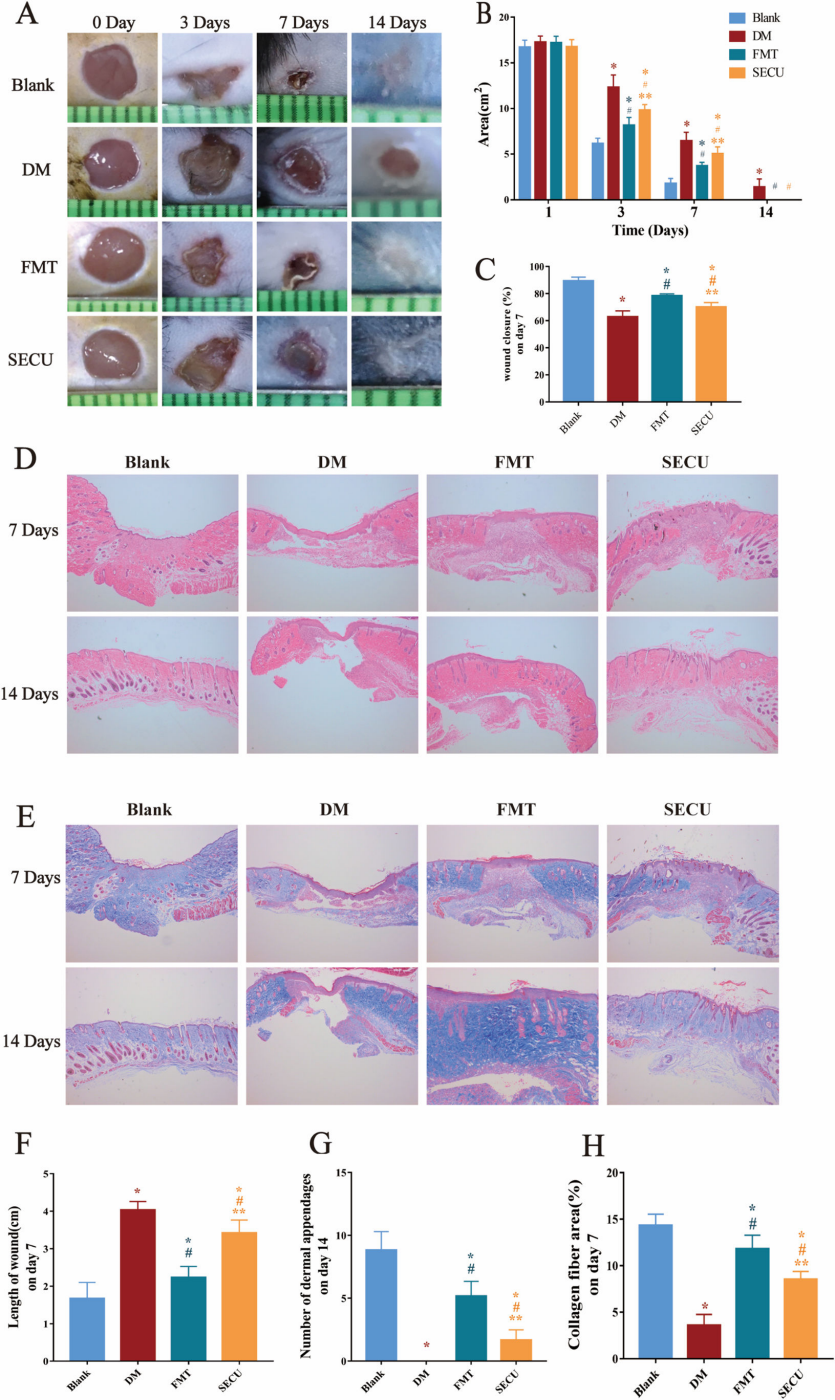
Wound-healing charts on days 0, 3, 7, and 14 in each group (**A**); wound areas (**B**); wound-healing rates on day 7 in each group (**C**); HE staining on days 7 and 14 in each group (**D**) (×40 magnification); wound length measurement on day 7 (**F**); and number of dermal appendages on day 14 (**G**). Masson staining of the skin wounds of mice in each group on days 7 and 14 (**E**) (×40 magnification) and the proportion of collagen fibers in the wounds on day 7 (**H**). Compared with blank, *P < 0.05; compared with DM, ^#^*P* < 0.05; compared with FMT, **P < 0.05.

After establishing a T2DM mouse model, a circular wound measuring 5 mm in diameter was created on the back (Fig. 1A), and the wound area was documented and assessed with ImageJ (Fig. 1B). As time elapsed, the wounds in all mouse groups showed gradual healing, but T2DM mice did not achieve complete healing until the 14th day. On the 3rd, 7th, and 14th days following the creation of the wound, compared with those of the normal mice, the wound areas of the T2DM mice were larger (*P < 0.05, Fig. 1B); following FMT, the wound areas in the mice were reduced in size compared to those in the DM group (^#^*P* < 0.05, Fig. 1B); and following administration of the IL-17A inhibitor secukinumab (SECU), the wound area in the T2DM mice from the SECU group was greater than that observed in the FMT group (**P < 0.05, Fig. 1B), yet still smaller than that in the DM group (^#^*P* < 0.05, Fig. 1B). The wound-healing rate of mice on the seventh day after wound formation was calculated. The wound healing of mice in the DM group was the slowest (*P* < 0.05, Fig. 1C), suggesting that the pathological conditions in the T2DM mice led to slower wound healing. After FMT, the wound healing in the T2DM mice was quicker than that observed in the DM group (^#^*P* < 0.05, Fig. 1C) but slower than that in the blank group (*P < 0.05, Fig. 1C), indicating that FMT has the potential to enhance wound healing in T2DM mice but cannot fully restore them to the level of normal mice. After administration of the IL-17A inhibitor SECU, wound healing in the SECU group was slower than that in the FMT group (**P < 0.05, Fig. 1C) but faster than that in the DM group (^#^*P* < 0.05, Fig. 1C). This indicates that the positive impact of FMT on healing in the T2DM mice was partially hindered by SECU, thereby demonstrating that part of the mechanism of FMT in promoting wound healing in T2DM mice is related to IL-17A.

### FMT changed the histological characteristics of wounds in T2DM mice

By comparing the hematoxylin and eosin (HE) staining (Fig. 1D) and Masson staining (Fig. 1E) images of each group of mice, the impact of FMT on the morphologies of mouse skin tissues was evaluated. The wound length on the 7th day after the wound was created was measured using ImageJ (Fig. 1F), and the number of dermal appendages on the 14th day following wound creation was statistically analyzed (Fig. 1G) using ImageJ. The proportion of collagen fibers on the seventh day after wound establishment was calculated using ImageJ (Fig. 1H). On the seventh day following the formation of the wound, compared with the DM group, the FMT group presented more granulation tissues in the mouse skin, with greater thickness and more layers (Fig. 1D), and had relatively abundant and well-organized collagen fibers, the quantity of which increased with the healing time (Fig. 1E). These findings suggest that FMT can encourage the generation of granulation tissues and collagen fibers after wound formation, but this effect can be inhibited by SECU. On the seventh day after wound formation, compared with normal mice, the wound length of the T2DM mice was larger (*P < 0.05, Fig. 1F); after FMT, the wound length of the mice was smaller than that of the DM group (^#^*P* < 0.05, Fig. 1F). After administration of the IL-17A inhibitor SECU, the wound length of the T2DM mice in the SECU group was larger than that of the FMT group (**P < 0.05, Fig. 1F), indicating that FMT can facilitate wound repair in T2DM mice. On the 14th day after wound formation, compared with the blank group, the number of dermal appendages in the T2DM mice was reduced (*P < 0.05, Fig. 1G); after FMT, the number of dermal appendages in the mice was greater than that in the DM group (^#^*P* < 0.05, Fig. 1G). On the seventh day after wound formation, the proportion of collagen fibers in the T2DM mice was the lowest (*P* < 0.05, Fig. 1H); after FMT, the percentage of collagen fibers was greater than that observed in the DM group (^#^*P* < 0.05, Fig. 1H), implying that FMT can promote skin repair, the regeneration of appendages, and the generation of wound collagen fibers, which are beneficial for the complete restoration of the skin.

### FMT regulates the imbalance of intestinal microbiota in T2DM mice

**Fig 2:**
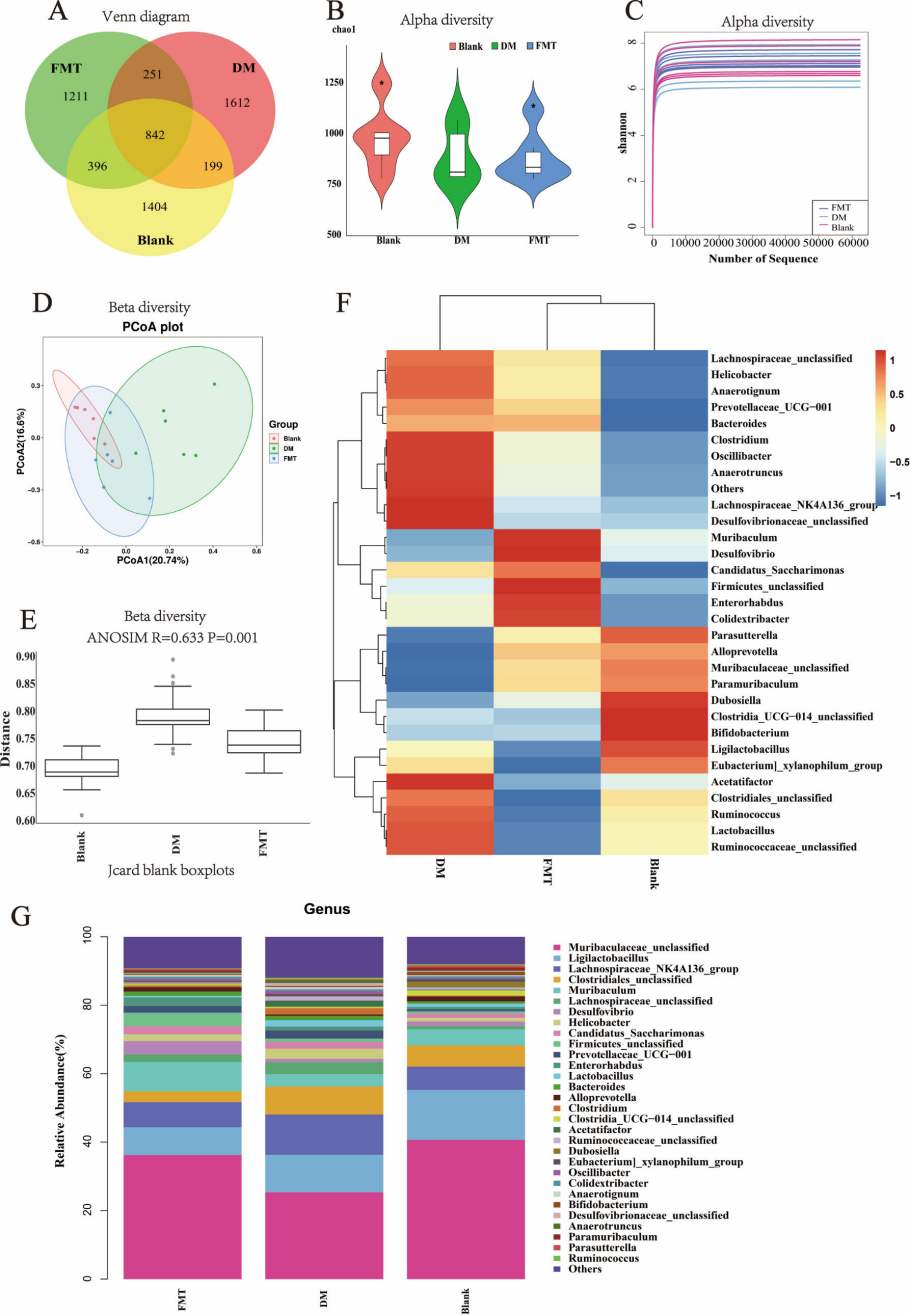
Fecal 16S rDNA sequencing results after microbiota transplantation in each group of mice. (A) Venn plot; (B) alpha diversity Chao1 index; (C) alpha diversity Shannon index; (D) beta diversity, principal coordinate analysis Bray_Curtis_PCoA plot; (E) beta diversity, similarity analysis Jaccard–ANOSIM plot; (F) cluster heat map; (G) column stacked plot of genus level abundance TOP30 species annotation.

**Fig 3:**
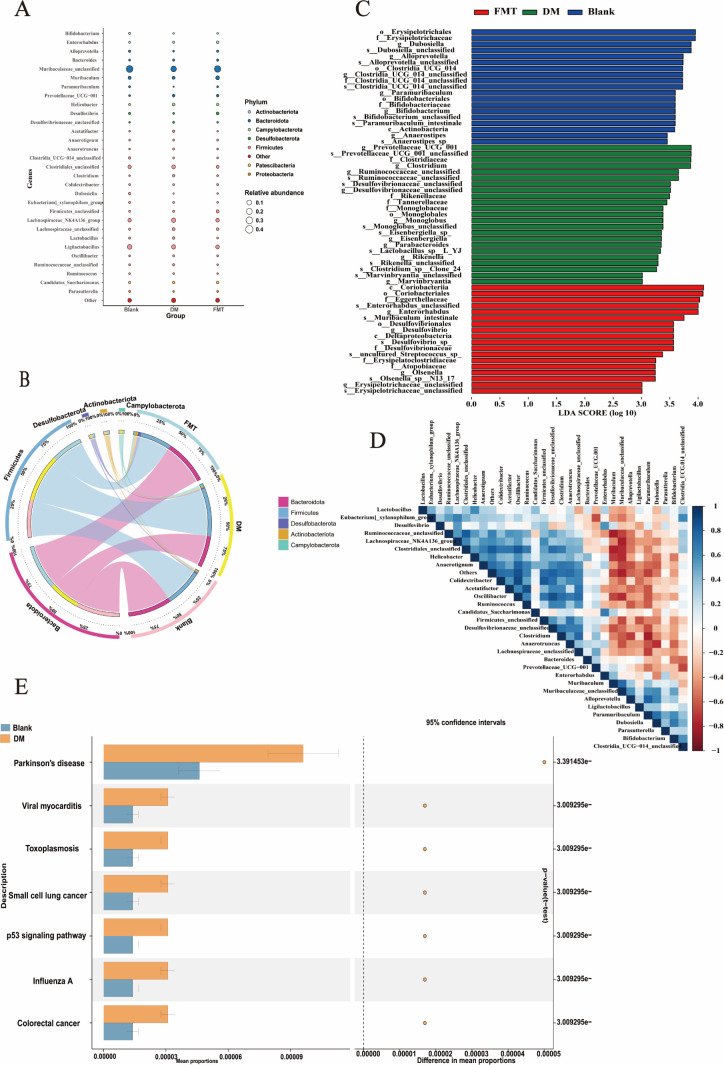
Advanced analysis of fecal 16S rDNA sequencing results after microbiota transplantation in each group of mice. (A) species composition bubble plot. (B) Species composition circle plot. (C) Biomarker screening (linear discriminant analysis [LDA] effect size) analysis. (D) Heatmap of the correlations of the top 30 microorganisms at the genus level in terms of abundance. (E) PICRUSt2 functional prediction plot.

Following FMT, 16S rDNA microbiota sequencing was conducted on the mouse excrement in the blank, DM, and FMT groups. Each group of samples underwent error correction for amplicon sequencing errors through QIIME2-based filtering, dereplication, and chimera removal. The abundance of unique feature sequences was quantified to generate amplicon sequence variant (ASV) tables, which facilitated the identification of true biological variations. As illustrated in the Venn diagram (Fig. 2A), the DM group shared 1,041 ASVs with the blank group while possessing 1,612 unique ASVs. In contrast, the FMT group shared 1,238 ASVs with the blank group and had 1,211 unique ASVs. These results suggest that the gut microbiota of T2DM mice exhibited significant alterations, whereas FMT treatment mitigated these changes. The Chao1 index of alpha diversity indicated that the number of species contained in the blank and FMT groups was greater than that in the DM group (*P < 0.05, Fig. 2B). The Shannon index also indicated that the blank and FMT groups were greater than the DM group (Fig. 2C). The Bray_Curtis_PCoA of Beta diversity plot (Fig. 2D) indicated that after FMT in the T2DM mice, their microbial composition structure was more similar to that of healthy mice, with smaller differences. By utilizing analysis of similarities (ANOSIM), the calculation result of the test statistic (*R* value) was 0.633, suggesting that significant variations existed among the groups. Meanwhile, the smaller the intragroup differences, the better the grouping effect. *P* = 0.001 suggested that a statistically significant difference existed and the grouping was meaningful (Fig. 3E).

Further analysis was carried out on the species annotation and prevalence of the intestinal microbiota at the genus level. The abundances of the top three genera (Fig. 2G) for the blank, FMT, and DM groups were 40.71, 36.27, and 25.36 (*Muribaculaceae_unclassified*); 14.59, 8.14, and 10.97 (*Ligilactobacillus*); and 6.76, 7.28, and 11.79 (*Lachnospiraceae_NK4A136_group*), respectively. In the distribution trend, the FMT group was more consistent with the blank group. Figure 2A illustrates the distribution of the top 30 microbiota at the genus level in each group and the corresponding information at the phylum level. The top three most prevalent species at the phylum level were *Bacteroidota* (50.09, 51.11, and 35.50), *Firmicutes* (42.67, 35.90, and 54.76), and *Desulfobacterota* (1.60, 3.39, and 1.51). The grouping information of the microbiota at the phylum level demonstrated that the microbiome of the FMT group of T2DM mice was closer to that of healthy mice and was opposite to that of the DM group of T2DM mice. Based on the abundance distribution of taxonomic units or the similarity degree among samples for clustering (Fig. 2F), the resemblance between the FMT and the blank groups was greater, while the disparity between the DM group and the other two groups was more pronounced, implying that the distribution of intestinal microbiota in the T2DM mice after FMT was closer to that of normal mice.

The detailed data (Table 1) showed that different microorganisms had a relative abundance exceeding 0.01% and a significance level lower than 0.05 at the genus level. In the DM group of T2DM mice, *Alloprevotella* (*P* = 0.0039), *Desulfovibrio* (*P* = 0.0374), and *Paramuribaculum* (*P* = 0.0037) were reduced significantly. However, after FMT treatment in the T2DM mice, *Alloprevotella* (*P* = 0.0374), *Desulfovibrio* (*P* = 0.025), and *Paramuribaculum* (*P* = 0.0037) rebounded. No notable difference was observed between the FMT and the blank groups in terms of *Alloprevotella* (*P* = 0.63) and *Desulfovibrio* (*P* = 0.2002), while the FMT group had a slightly lower *Paramuribaculum* (*P* = 0.02) level than the blank group. In the DM group, the T2DM mice showed significantly increased *Prevotellaceae_UCG-001* (*P* = 0.02), *Clostridium* (*P* = 0.0039), and *Desulfovibrionaceae_unclassified* (*P* = 0.02) compared with the blank group. After FMT treatment, *Prevotellaceae_UCG-001* (*P* = 0.04), *Clostridium* (*P* = 0.0374), and *Desulfovibrionaceae_unclassified* (*P* = 0.02) returned to lower levels. Furthermore, linear discriminant analysis effect size (LEfSe) was performed to determine the species exhibiting notable variations in abundance across the groups (Fig. 3C), and the potential biomarkers of each group were screened. At the genus level, for the blank group, they were *Dubosiella* and *Alloprevotella*; for the DM group, they were *Prevotellaceae_UCG-001* and *Clostridium*. In the FMT group, the biomarkers corresponding to the microbiota of T2DM mice in the blank group were relatively elevated, while those corresponding to the DM group declined relatively, indicating that there was a similar microbiome profile between the FMT and the blank groups. The correlation analysis shows that *Dubosiella* shows an affirmative relationship with *Alloprevotella* and an inverse relationship with *Prevotellaceae_UCG-001* and *Clostridium*, while *Prevotellaceae_UCG-001* and *Clostridium* are positively correlated with each other. Based on the functional prediction results of Phylogenetic Investigation of Communities by Reconstruction of Unobserved States (PICRUSt2) (Fig. 3E), according to the annotation results of the Statistical Analysis of Metagenomic Profiles (STAMP) differential analysis of the Kyoto Encyclopedia of Genes and Genomes (KEGG) database function with *P* < 0.05, a total of seven level 3 pathways were identified, which were related to Parkinson’s disease (PD), viral myocarditis, toxoplasmosis, small cell lung cancer, p53 signaling pathway, influenza A, and colorectal cancer.

**TABLE 1:** Proportional abundance of microbiota at the genus level^*a*^

Annotation of species (genus)	Relative abundance (%)			*P* value			
	Blank	DM	FMT	Overall	B vs D	F vs D	B vs F
*Alloprevotella*	1.25	0.37	1.42	0.01	0.0039	0.0374	0.63
*Desulfovibrio*	3.78	1.01	1.35	0.11	0.0374	0.025	0.2002
*Paramuribaculum*	0.42	0.03	0.82	0.0009	0.0037	0.0037	0.02
*Prevotellaceae_UCG-001*	1.93	2.44	0.60	0.01	0.02	0.04	0.0039
*Clostridium*	0.56	1.84	0.27	0.01	0.0039	0.0374	0.0021
*Desulfovibrionaceae_unclassified*	0.25	0.84	0.23	0.04	0.02	0.02	0.87

^*a*^ Statistical evaluation was conducted using the Kruskal–Wallis multiple comparison test followed by the Mann–Whitney *U* test for post hoc analysis.

Following the successful establishment of the T2DM model, the sequencing results of fecal microbiota in the T2DM mice revealed significant differences across both the phylum and genus classifications compared to healthy mice. These findings suggest an imbalance in bacterial composition among T2DM mice, which was significantly reduced following FMT.

### FMT enhances the upregulation of IL-17A expression in T2DM mice

**Fig 4:**
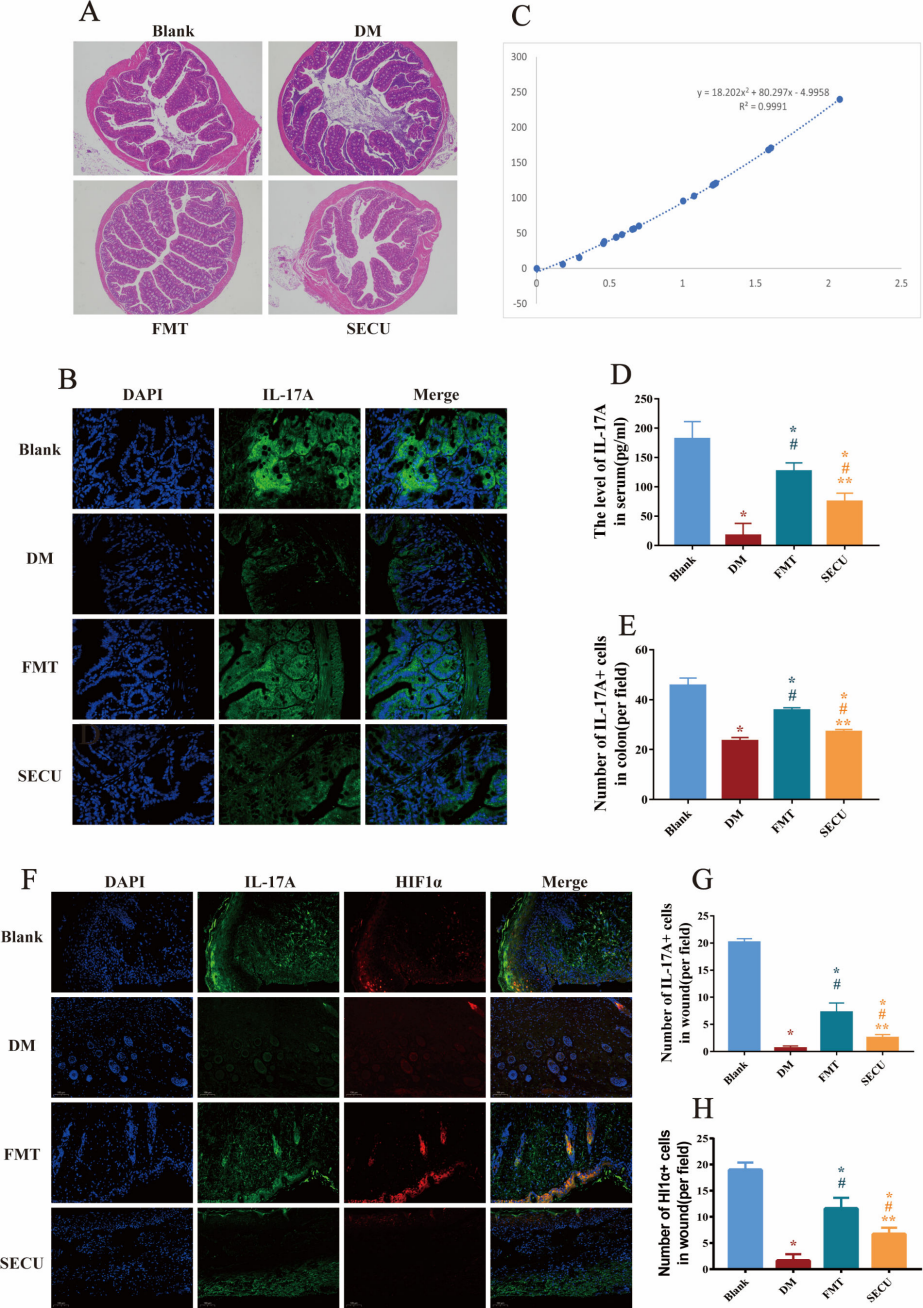
HE staining (**A**) (×40 magnification) and immunofluorescence staining (**B**) (×100 magnification) of the intestinal tract of the mice within each group on day 14 of wound healing. ELISA showed IL-17A content in mouse serum (**C**) and its statistical bar chart (**D**), and the quantity of IL-17A-positive cells in the intestinal tract of the mice from each group as observed under an immunofluorescence staining microscope (**E**). Immunofluorescence staining of the skin of mice in each group on the 14th day of wound healing (**F**) (×100 magnification); IL-17A-positive cells (**G**) and HIF1α-positive cells (**H**) under the immunofluorescence staining microscope. Compared with blank, *P < 0.05; compared with DM, ^#^*P* < 0.05; compared with FMT, **P < 0.05. DAPI, 4′,6-diamidino-2-phenylindole.

On the 14th day following the wound creation, the mouse colons were collected. HE staining and immunofluorescence staining were conducted on the colonic tissues. The findings indicated no significant alterations within the colonic architecture of the mice in each group (Fig. 4A). Nevertheless, the immunofluorescence results (Fig. 4B and E) revealed that the quantity of IL-17A-positive cells in the DM group decreased (*P < 0.05, Fig. 4E). After FMT, the quantity of IL-17A-positive cells increased (^#^*P* < 0.05, Fig. 4E); however, it remained reduced (*P < 0.05, Fig. 4E). Following the administration of SECU, the number of IL-17A-positive cells decreased once again (**P < 0.05, Fig. 4E). This indicated that the colon structure of the T2DM mice was not impaired. The amount of IL-17 in the colons of the T2DM mice was comparatively lower than that in the blank group following wound formation, and FMT increased the concentration of IL-17A in the colons of the T2DM mice to nearly that of the normal mice. The content of IL-17A in the mouse serum on day 14 after wound formation was detected by enzyme-linked immunosorbent assay (ELISA) (Fig. 4C and D). The IL-17A in the serum of the T2DM mice was markedly diminished (*P < 0.05, Fig. 4D), and after FMT, the IL-17A content increased (^#^*P* < 0.05, Fig. 4D). Immunofluorescence staining on the 14th day (Fig. 4F) after wound formation indicated that the levels of IL-17A and HIF1α expression in the skin of the DM mice reduced (*P < 0.05, Fig. 4G and H) but increased after FMT (^#^*P* < 0.05, Fig. 4G and H). After inhibition of IL-17A (**P < 0.05, Fig. 4G), HIF1α also decreased (**P < 0.05, Fig. 4H). The results demonstrated that the amounts of IL-17A in the colon, serum, and skin of the T2DM mice on day 14 post-wound formation considerably reduced in comparison to those in the blank group. FMT treatment in the T2DM mice led to the elevation of IL-17A in the colon, serum, and skin; however, this effect was attenuated by SECU administration. Additionally, a decrease in HIF1α expression was observed, suggesting a correlation between HIF1α expression and IL-17A levels in the derma.

**Fig 5:**
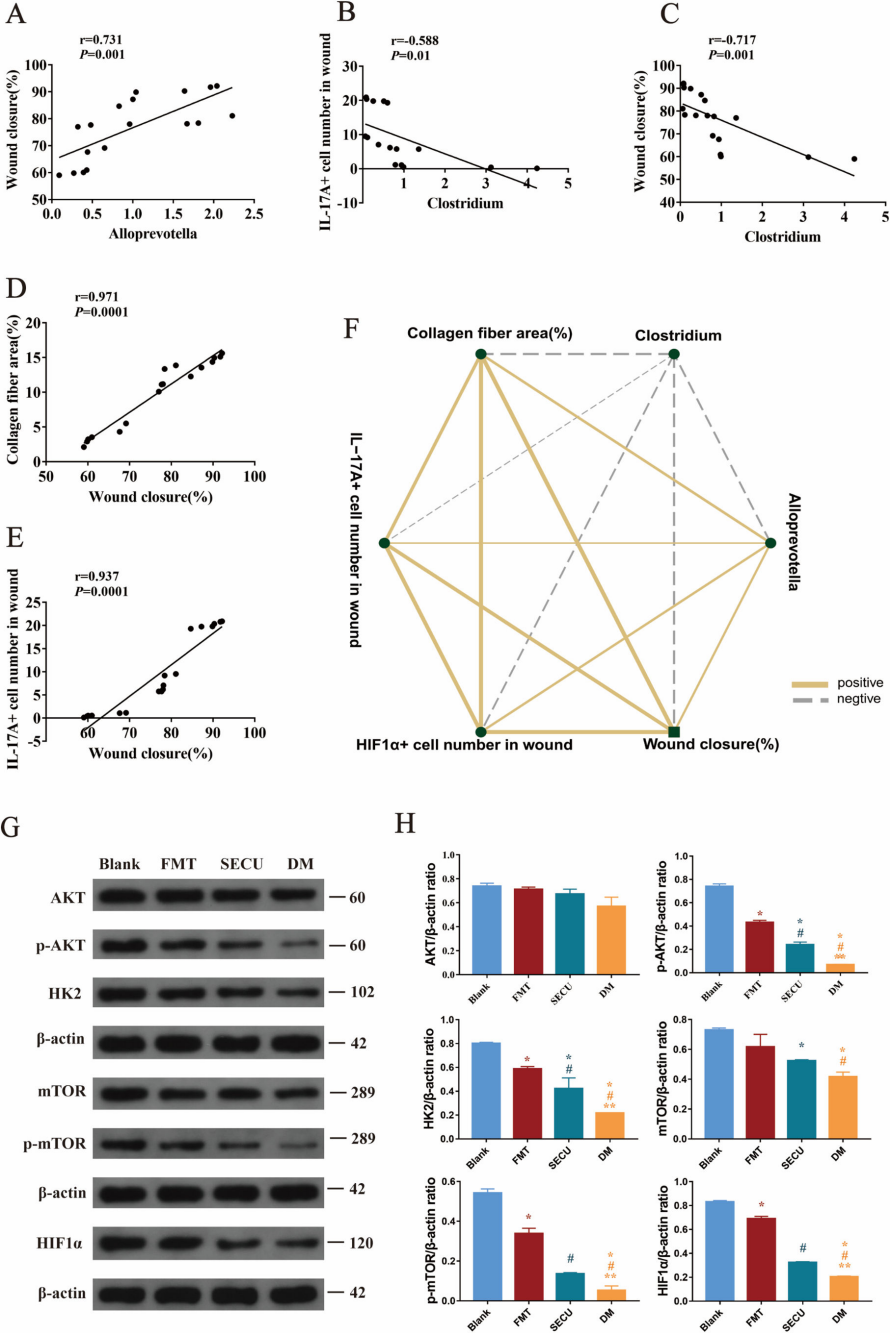
Association between *Alloprevotella* and wound closure (%) (**A**). Correlation analysis between *Clostridium* and IL-17A+ cell number in the wound (**B**), correlation analysis between *Clostridium* and wound closure (%) (**C**), analysis of the correlation between wound closure (%) and collagen fiber area (%) (**D**), analysis of the correlation between wound closure (%) and IL-17A+ cell number in the wound (**E**), and correlation network diagram between each indicator (**F**). The solid line represents a favorable relationship, while the dashed line signifies an inverse relationship. The width of the line indicates the strength of the correlation. A positive *R* value signifies a positive correlation. *P* < 0.05 is considered statistically significant. Western blot detection of pathway proteins in the skin of each group of mice on the 14th day (**G**) and their statistical results (**H**). Compared with blank, *P < 0.05; compared with FMT, ^#^*P* < 0.05; compared with SECU, **P < 0.05.

*Alloprevotella* was recognized as a potential biomarker for the blank group, while *Clostridium* showed promise as a biomarker for the DM group. Analysis of correlation (Fig. 5) showed negative associations between *Alloprevotella* and *Clostridium* but positive correlations with wound closure rate, collagen fiber area, IL-17A-positive cell count within wounds, and HIF1α-positive cell count within wounds among the experimental groups. Following FMT treatment, there was an increase in *Alloprevotella* abundance accompanied by a reduction in *Clostridium* levels, which positively correlated with the IL-17A expression, production of collagen fibers within the skin tissue, and enhanced wound-healing capacity. These findings collectively indicate that FMT has potential therapeutic benefits for promoting wound-healing processes specifically tailored to T2DM mice.

### IL-17A promotes the migration of human keratinocytes

**Fig 6:**
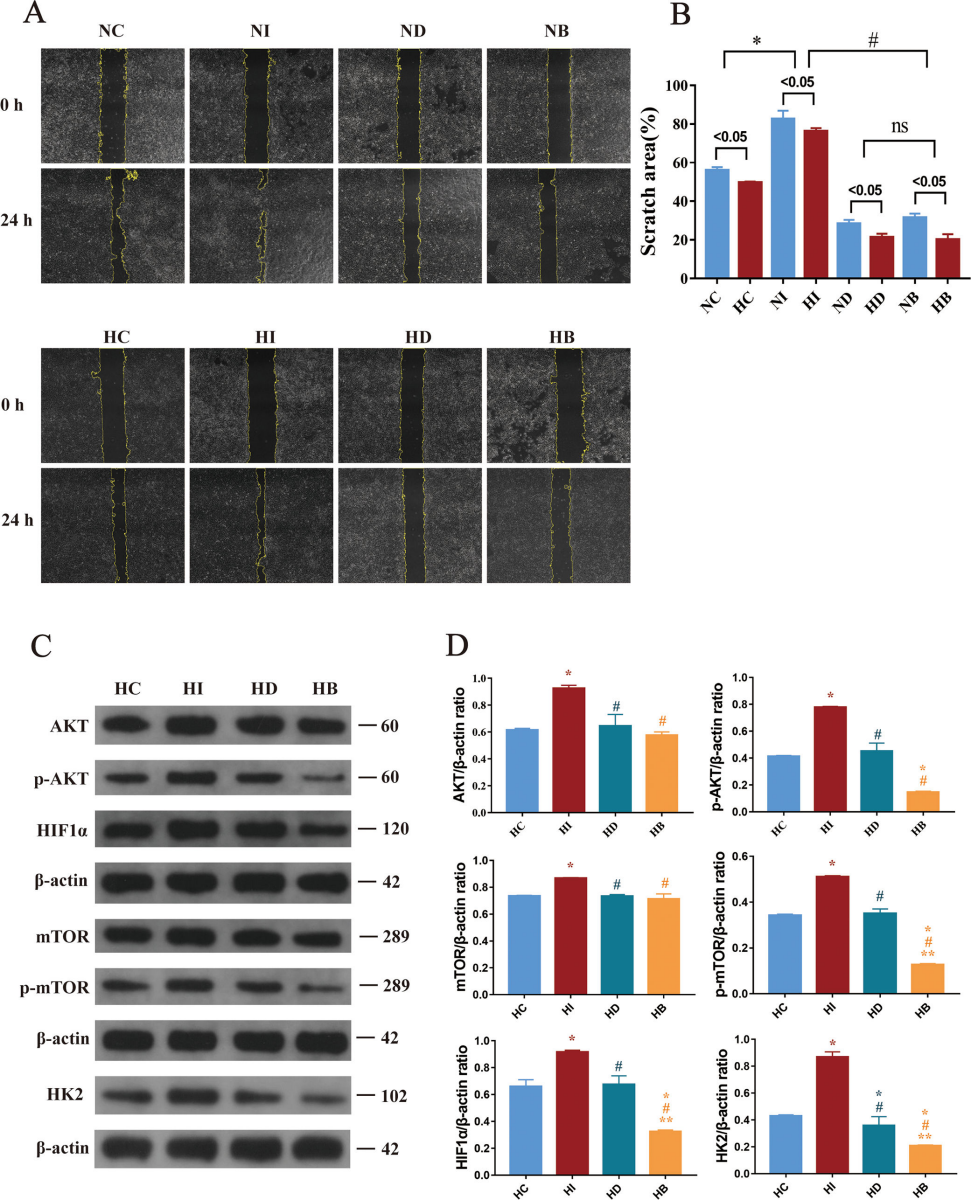
Experimental records of human keratinocytes in each group at 0 and 24 h after scratch (**A**) (×100 magnification) and comparison of their mobility (**B**). Western blot detection of pathway proteins in cells (**C**) and their statistical results (**D**). Compared with HC, *P < 0.05; compared with HI, ^#^*P* < 0.05; compared with HD, **P < 0.05.

Human keratinocytes were cultured, and cell scratch assays were conducted. The results are illustrated in Fig. 6. The area of cell migration in the high-glucose medium group was considerably lower than that in the control group (*P* < 0.05, Fig. 6A and B). Subsequently, comparisons involving pharmacological agents were performed. Following the addition of exogenous IL-17A, a significant increase in cell migration area was noted in the NI and HI groups when compared to the control group (*P < 0.05, Fig. 6B). Conversely, treatment with the glycolysis inhibitor 2-deoxy-D-glucose (2-DG) in the ND groups and HD groups and HIF1α inhibitor BAY87-2243 in the NB groups and HB groups led to a reduction in cellular movement area (*P < 0.05, ^#^*P* < 0.05, Fig. 6B), with no notable discrepancy between the two inhibitor groups. These findings indicate that IL-17A promotes keratinocyte migration, while inhibition of HIF1α and glycolysis suppresses this process.

### IL-17A upregulates the expression of the IL-17A–mTOR–HIF1α signaling pathway and facilitates glycolysis

Skin tissues were collected from the sacrificed mice. Human keratinocytes were harvested at 24 h, followed by protein extraction and Western blot (WB) analysis. Regarding the WB results of mouse skin tissue (Fig. 5G and H), compared with the blank group, the expression levels of p-AKT, p-mTOR, and HIF1α in the DM group were significantly lower (*P < 0.05, Fig. 5H). In contrast, these protein levels were higher in the FMT group (^#^*P* < 0.05, Fig. 5H). Additionally, analysis of IL-17A expression in the colon, serum, and skin revealed that IL-17A levels in the FMT-treated diabetic mice were elevated compared to untreated diabetic mice (Fig. 4). This increase in IL-17A led to the activation of AKT and mTOR signaling proteins, enhanced HIF1α expression, and increased the expression of HK2, a rate-limiting enzyme in glycolysis, thereby promoting wound healing. Further validation of these findings was conducted in human keratinocytes (Fig. 6C and D). In contrast to the control, intervention with IL-17A resulted in the activation of AKT and mTOR proteins, an upregulation of HIF1α expression, and a concomitant increase in the expression of HK2, an essential rate-limiting enzyme in glycolysis (*P < 0.05, Fig. 6D). Inhibition of HIF1α and glycolysis led to decreased downstream expressions of both HIF1α and HK2, indicating that IL-17A activates mTOR via the AKT signaling pathway, enhances HIF1α expression, promotes glycolysis, and consequently accelerates keratinocyte migration.

## DISCUSSION

The integration of high-fat diets and low-dose streptozotocin (STZ) currently constitutes the most prevalently applied method for establishing T2DM models. In keeping with the relevant literature (26) and the outcomes of pre-experimental studies, mice were given a high-fat diet for 4 weeks, after which they received intraperitoneal injections of STZ at a dosage of 45 mg/kg/48 h. The fasting blood glucose (FBG) levels measured from the tail veins of the mice were all greater than 11.1 mM. Hence, the success of the T2DM mouse model was determined based on the fasting blood glucose level. Subsequently, a circular wound measuring 5 mm in diameter was made on the backs of T2DM mice to assess the recovery process of wounds in diabetics.

This study is the first to observe the facilitating effect of FMT on the healing of diabetic wounds. There are many reasons for the difficulty in healing T2DM wounds. Elevated serum glucose induces damage to multiple cell types, including endothelial cells, neurons, keratinocytes, and fibroblasts (27). Diabetic neuropathy and vascular disorders also affect skin healing. The intestinal microbiome is regarded as an essential “organ” for boosting host immunity, aiding food digestion, regulating intestinal endocrine functions, modulating neural signal transduction, altering metabolism, and eliminating toxins. After performing healthy FMT in T2DM mice in this experiment, the wound area of the FMT group was reduced on the 3rd, 7th, and 14th days; the wound healing improved; and the length of the wound decreased. From the histological characteristics, we determined that after FMT, the skin migration of the T2DM mice was accelerated; there was more granulation tissue; the recovery of dermal appendages increased; and the generation of collagen fibers increased. These observations suggested that the facilitating effect of FMT on wound healing is associated with the increased development of granulation tissue, the accelerated migration of keratinocytes, and the augmented production of collagen fibers by fibroblasts. The specific mechanism awaits further investigation.

FMT involves the transfer of gut microbiota from healthy mice to the intestines of T2DM mice and serves as a natural method for correcting imbalances in gut microbiota. Such imbalances are associated with the development of T2DM and can affect glucose and lipid metabolism (28), and they are strongly connected to the development of obesity. About 86% of individuals with T2DM are either overweight or obese, making obesity the most significant contributing factor for T2DM (29). Clinically, it has also been confirmed that T2DM patients have a gut microbiota imbalance, with reduced diversity and altered abundance of gut microbiota (30). Therefore, regulating the gut microbiota imbalance in T2DM through FMT holds significant application prospects.

As shown in the Venn diagram (Fig. 2A), each group of ASV, after background noise removal, not only significantly enhances data accuracy and species resolution but also ensures the robustness and reliability of the results (31). The number of shared ASV between the DM group and the blank group was lower than that between the FMT group and the blank group. The DM group exhibited the highest number of unique ASVs, indicating significant alterations in the gut microbiota of diabetic mice. These variations can be mitigated by FMT treatment. Alpha diversity measures the diversity within a specific environment or ecosystem. The Chao1 and Shannon indices (Fig. 2B and C) decreased in the T2DM group, illustrating the decline in the total number of species, the unknown factors, and uncertainties within this community. ANOSIM indicates substantial intergroup differences and minimal intragroup variation, which suggests effective grouping of this study. The *P* value of 0.001 indicates statistically significant differences, confirming that the grouping is meaningful. Principal coordinate analysis (PCoA) (Fig. 2D) suggests that the species composition structure of T2DM mice differs significantly from that of healthy mice, while FMT can reduce this disparity, bringing the microbial composition of diabetic mice closer to that of healthy controls. There are complex and bidirectional relationships between diabetes and the gut microbiota (32). The gut microbiota and its metabolites can influence insulin sensitivity (13), and a well-balanced microbiota ecosystem may contribute to the alleviation of diabetes (33). Fecal microbiota alterations may be associated with the onset and development of diabetes, whereas FMT can alleviate this change, which suggests that FMT may offer a potential therapeutic approach for improving diabetes and its associated complications.

The primary objective of LEfSe analysis is to identify biomarkers, specifically species with significant abundance differences among multiple groups. It provides a more intuitive visualization of these differences across all taxonomic levels between groups and is currently one of the most frequently used methods for displaying differential species. At the genus level, mice in the DM group exhibited significant decreases in *Alloprevotella*, *Desulfovibrio*, and *Paramuribaculum*, while *Prevotellaceae_UCG-001*, *Clostridium*, and *Desulfovibrionaceae_unclassified* increased significantly. The decrease in *Desulfovibrio* can facilitate the development of glucose and lipid metabolic disorders (34). *Prevotellaceae_UCG-001* increases in diseases such as colitis (35), depression (36), and aging (37) and is regarded as a harmful bacterium. *Clostridium* is a key intestinal microorganism that affects the production of secondary bile acids (38), thereby affecting lipid digestion and absorption. *Desulfovibrionaceae_unclassified* has been found to increase in the intestines of mice fed a high-fat diet and is an LPS-associated bacterium related to intestinal inflammation (39). The potential biomarkers of healthy mice, such as *Dubosiella*, *Paramuribaculum*, and *Alloprevotella*, also significantly decreased in the DM group. *Dubosiella* facilitates the establishment of an intestinal immune microenvironment in mice (40), and it shows potential in slowing down the progression of Alzheimer’s disease by producing palmitoleic acid, a compound with protective effects against inflammation and metabolic disturbances (41). *Paramuribaculum* can produce butyrate and enhance blood glucose management (42), and butyrate controls immune regulation, promotes the integrity of the intestinal epithelium, regulates insulin secretion and pancreatic B-cell proliferation, and plays multiple roles in T2DM (43). *Alloprevotella* assists in the digestion of fibers into short-chain fatty acids, and its reduction can influence the digestion of fibers (44). Therefore, *Alloprevotella*, *Desulfovibrio*, *Paramuribaculum*, and *Dubosiella* and their metabolic products, such as palmitoleic acid, short-chain fatty acids, and butyrate, may play beneficial roles in the healing of diabetic wounds, which requires further in-depth investigation. In this study, after FMT was performed on T2DM mice, the abundance of *Alloprevotella*, *Desulfovibrio*, *Paramuribaculum*, and *Dubosiella* at the genus level increased relatively, while that of *Prevotellaceae_UCG-001*, *Clostridium*, and *Desulfovibrionaceae_unclassified* decreased, indicating that FMT restored the microbiota diversity in T2DM mice, causing the microbiota structure to approach that of normal healthy mice, increasing the advantageous bacteria while decreasing the deleterious bacteria, thereby addressing the imbalance in the intestinal microbiota in T2DM mice. Nevertheless, this study focused solely on the beneficial effects of FMT from healthy mice on diabetic mice. In future clinical applications, individual variability among patients and differences in their microbiota will inevitably play a significant role. In this study, building on the findings that FMT positively influenced *Alloprevotella*, *Desulfovibrio*, *Paramuribaculum*, and *Dubosiella* in diabetic mice, further research is warranted to systematically identify and refine beneficial bacterial strains from healthy individuals for diabetic patients. By conducting personalized microbiota sequencing for diabetic patients, targeted transplantation of deficient bacterial species can be performed. This approach promises to enhance treatment efficacy and advance precision medicine.

PICRUSt establishes a “mapping” between the microbiota and their functional profiles, making it an indispensable tool for predicting microbiota functions (45). The STAMP analysis results presented those functions with *P* values of <0.05 from pairwise *t*-tests. A total of seven level 3 pathways were identified, which were related to PD, viral myocarditis, toxoplasmosis, small cell lung cancer, p53 signaling pathway, influenza A, and colorectal cancer. Observational studies suggest that individuals with T2DM may face a higher risk of developing PD. These effects could be influenced by shared cellular mechanisms (46). Based on the PICRUSt functional prediction of this study, it can be preliminarily speculated that the gut microbiota of T2DM mice is also related to PD. In the treatment of T2DM and PD, several common signaling pathways have been identified (46). Insulin can modulate neuroinflammation by interfering with NFκB through the PI3K/AKT/GSK-3β pathway (47). During Exenatide treatment for PD patients, it was observed that the Akt and mTOR signaling pathways were upregulated (48). However, the roles of these pathways in diabetic wound healing remain to be confirmed. These findings further highlight the significant potential of FMT.

The experimental outcomes demonstrated that FMT not only regulates the microbiota imbalance in T2DM mice and promotes the healing of diabetic wounds but also enhances IL-17A in the mouse colon, serum, and skin. Moreover, the analysis of correlations indicated that the expression of *Alloprevotella* positively correlates with that of IL-17A, while the expression of *Clostridium* negatively correlates with IL-17A, implying that the alterations in microbiota are linked to the variations in IL-17A. Research (49) has revealed that transplantation of healthy intestinal microbiota can facilitate the expression of IL-17A dependent on microorganisms, which is conducive to restoring the intestinal barrier in alcoholic liver disease. The transplantation of fungi in the healthy intestinal microbiota can boost the expression of IL-17A throughout the mouse body and promote intestinal barrier function (19). An experiment on muscle injury in mice indicated that the production of IL-17A by γδT cells occurs in a microbe-dependent manner, and simultaneously, IL-17A can accelerate the repair of injuries (50). These are all in line with the results of this experiment, suggesting that the transplantation of healthy microbiota can stimulate the generation of IL-17A. IL-17A exerts its influence through interaction with IL-17RA and IL-7RC, and IL-17 receptors are distributed in various cells, including keratinocytes (51), epithelial cells (52), and fibroblasts (53). An increasing body of research indicates that IL-17A is essential for the processes of injury repair (50), fibrogenesis (53), and skin healing (54), and both skin fibrogenesis and wound healing are related to the HIF1α signaling pathway (53, 54). The results of this experiment indicated that after FMT in T2DM mice, the levels of IL-17A expression rose; the generation of collagen fibers throughout the recovery phase of diabetic wounds increased; and the wound healing accelerated. The correlation results indicated that IL-17A positively correlates with collagen fiber deposition and the rate of wound healing.

Further studies on the potential mechanisms were carried out by culturing human keratinocytes. The results of the scratch assay indicated that after the addition of exogenous IL-17A, the migration rate of keratinocytes was significantly accelerated. However, when HIF1α and glycolysis were inhibited, the migration rate of keratinocytes decreased, suggesting that the function of IL-17A in promoting cell migration is via the HIF1α and glycolysis pathways. The WB results indicated that the addition of IL-17A could activate the pathway protein AKT, which, in turn, activates mTOR, resulting in the upregulation of HIF1α and the expression of the glycolytic enzyme HK2. After the HIF1α inhibitor was added, no significant alterations were noted in the expressions of AKT and mTOR in the HB group, while HIF1α and HK2 significantly decreased, and cell migration decreased. Following the incorporation of the HK2 inhibitor, the expressions of the other proteins in the HD group were not affected, and only the expression of HK2 was reduced. This indicates that IL-17A improves the amount of HIF1α, enhances glycolysis, and thereby accelerates cell migration through the IL-17A–mTOR–HIF1α signal axis. Activated HIF-1α can control the transcriptional process of glycolysis (55), and it enhances intracellular glucose uptake and glycolysis while downregulating oxidative phosphorylation in mitochondria, thereby regulating the energy supply for wound epithelial cell migration (56, 57) on the wound surface and promoting cell migration (58), which is consistent with the results of this experiment.

The study demonstrated through mouse experiments that FMT can correct microbiota dysbiosis in T2DM mice, regulate IL-17A expression, and promote diabetic wound healing. Additionally, experiments on human keratinocytes revealed the positive role of the IL-17A signaling axis in cell migration. Future work will expand the sample size to evaluate long-term therapeutic effects and conduct a more comprehensive assessment of the role and mechanisms of gut microbiota and its metabolites in diabetic patients. Combined with clinical trials, these findings will provide a stronger foundation and direction for future clinical applications.

## MATERIALS AND METHODS

### Animal experiments

Four-week-old male C57BL/6J mice (weighing approximately 20 ± 3 g) were obtained from the Animal Laboratory Center of Zhejiang University, China. The mice underwent a 1-week acclimatization period in a controlled environment with a 12 h light and dark cycle. The temperature was maintained at 22°C ± 2°C, and the moisture level between 50% and 60%, with unrestricted availability of food and water.

### Mouse model of T2DM

A total of 24 mice were randomly allocated to four groups: the blank control group (blank group), the T2DM model group (DM group), the T2DM model group with microbiota transplantation (FMT group), and the IL-17A inhibitor group (SECU group). The DM, FMT, and SECU groups were utilized to create T2DM mouse models. Eighteen of these mice received a high-fat diet consisting of 20% protein, 20% carbohydrates, and 60% fat (Research Diets, D112252) (59). In contrast, the mice belonging to the blank group were given a standard diet and water. After 4 weeks, STZ was prepared in a 0.1 M citrate buffer at a pH of 4.5. The mice in the model groups received intraperitoneal injections of STZ (CS10491-100 mg, Coolaber) at a dosage of 45 mg/kg every 48 h, while the blank group was given an equivalent volume of citrate buffer via intraperitoneal injection (26). Following three intraperitoneal injections of STZ while the mice were fasting, FBG levels were assessed. An FBG level exceeding 11.1 mM was considered indicative of T2DM, and measurements were taken using a glucose meter (Omron, Japan).

### Fecal microbiota transplantation

**Fig 7:**
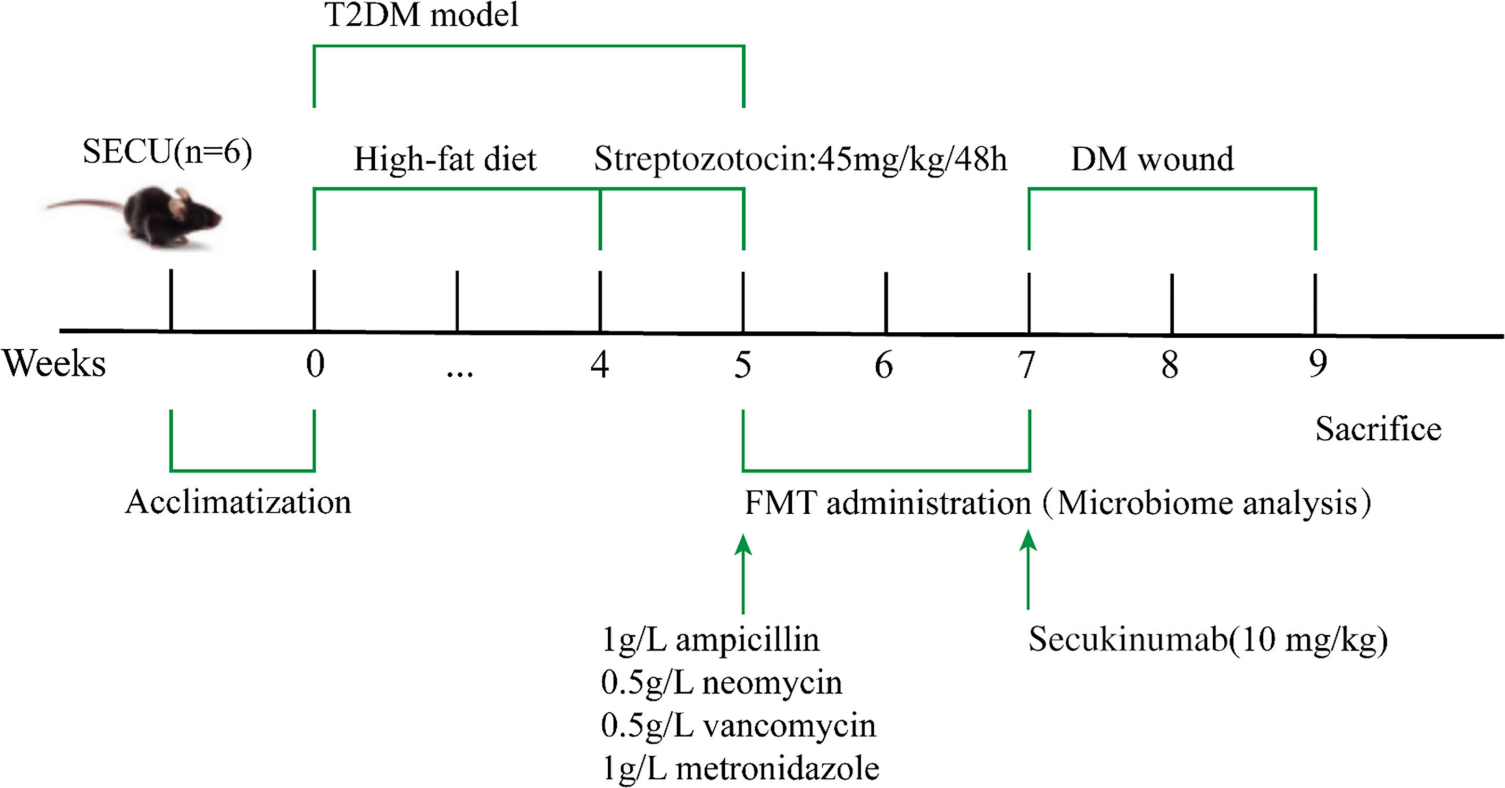
Schematic diagram of the procedure for the SECU group.

In the FMT and SECU groups, the mice received fecal microbiota transplants from healthy donors. Conversely, the blank and DM groups underwent blank transplantation. The first step of microbiota transplantation entailed administering 200 µL of antibiotic cocktail orally to mice for three consecutive days, comprising 1 g/L ampicillin, 0.5 g/L neomycin, 0.5 g/L vancomycin, and 1 g/L metronidazole (60). Before gavage, the mice fasted for 6 h. For blank transplantation, the same volume of normal saline was administered orally. Twenty-four hours after the administration of antibiotics, the FMT group and the SECU group were given 100 µL of freshly prepared fecal suspension from healthy mice, administered via gavage every 48 h for 2 weeks (Fig. 7). For blank transplantation, the same volume of phosphate-buffered saline (PBS) was administered orally. For the collection of the fecal suspension from healthy mice, all the healthy mice of the blank group were transferred to clean and sterile cages. Twelve fresh and clean feces from healthy mice (approximately 300 g) were collected into sterile and clean Eppendorf (EP) tubes. The feces were shaken and completely dissolved in 1,800 µL PBS by re-suspension. The solution was spun in a centrifuge at 2,500 rpm for 1 minute, and the upper suspension was collected. Then, 100 µL was administered orally to each mouse. Fresh feces were collected for each microbiota transplantation. Following the microbiota transplantation, fecal samples from mice in the blank, DM, and FMT groups were gathered for 16S rDNA sequencing.

### Diabetic wound-healing experiment

Once the mice were fully anesthetized through an intraperitoneal injection of 1% sodium pentobarbital at a dosage of 0.3 mg/kg, the fur on their backs was shaved and disinfected. A circular full-thickness skin wound measuring 5 mm in diameter was then made on the posterior back near the hip joint using a punch tool (61). After the creation of the wound, each mouse was housed separately in a single cage to prevent self-scratching. One hour prior to wound creation, as illustrated in Fig. 6, mice in the SECU group received a subcutaneous injection of 10 mg/kg of the IL-17A inhibitor SECU (S412013, Aladdin), while the remaining groups were administered an equivalent amount of sterile saline solution (62). The injection was administered once a week until sacrifice. Wound healing was assessed using ImageJ on days 0, 3, 7, and 14 after the wound was created. The wound closure (%) was calculated as (W_0_ – *W*_*n*_) / *W*_0_ × 100%; *W*_0_ and *W*_*n*_ indicate the wound area on days 0 and *n*. Two weeks later, the mice were sacrificed under general anesthesia, and colon, serum, and skin specimens were gathered for corresponding tests.

### Hematoxylin and eosin staining and Masson’s trichrome staining

Following general anesthesia, the mice were perfused through the heart with a 0.9% saline solution, followed by a perfusion of 0.1 M phosphate buffer with 4% paraformaldehyde. The colon and skin tissues were carefully excised and promptly placed in 4% paraformaldehyde for 24 h. These tissues were subsequently embedded in paraffin and cut into 5 µm sections. The embedding medium was removed using solvents such as xylene. The sections were re-hydrated with graded alcohol solutions. Hematoxylin and eosin were used for HE staining, and Weigert’s iron hematoxylin staining solution was employed for Masson staining. The experimental results were then observed under an optical microscope.

### Fecal 16S rDNA sequencing

Each mouse was placed in a separate sterile cage, and fresh fecal pellets were collected in sterile EP tubes. The samples were immediately chilled and stored at −80°C for subsequent analysis. Total DNA was extracted from microbial community samples using the cetyltrimethylammonium bromide method. DNA integrity was assessed by electrophoresis, and quantification was done with a UV-vis spectrophotometer. The V3–V4 (63) hypervariable region of the bacterial 16S rDNA gene was selected for PCR amplification. The forward primer 341F (5′-CCTACGGGNGGCWGCAG-3′) and the reverse primer 805R (5′-GACTACHVGGGTATCTAATCC-3′) were used. PCR products were confirmed via 2% agarose gel electrophoresis. Purification was performed using AMPure XT beads (Beckman Coulter Genomics), followed by quantification with Qubit (Invitrogen). Purified products were recovered using an AMPure XT bead recovery kit. The refined PCR products were evaluated using an Agilent 2100 Bioanalyzer and a Kapa Biosciences Illumina library quantification kit. A qualified sequencing library, with unique index sequences, underwent gradient dilution and proportional mixing based on required sequencing amounts. The library was denatured into single strands for sequencing on a NovaSeq 6000 sequencer, with paired-end sequencing at 2 × 250 bp using the NovaSeq 6000 SP Reagent Kit (500 cycles), followed by data analysis. Sequencing was performed on an Illumina NovaSeq platform. Paired-end reads were assigned to samples using unique barcodes and processed by removing barcode and primer sequences. The paired-end reads were assembled with FLASH. Raw reads were filtered to generate high-quality clean tags using fqtrim (v.0.94). Chimeric sequences were identified and removed with Vsearch (v.2.3.4). After dereplication with DADA2, we generated an ASV feature table and extracted feature sequences. Alpha and beta diversity metrics were computed based on randomly subsampled sequences to ensure equal sequence depth using QIIME2. Chao1 estimator estimates total species richness in a community. The Shannon index is derived from the information entropy of a community. The beta diversity was visualized through PCoA. ANOSIM is a non-parametric test that evaluates whether differences between groups, based on a Jaccard index-derived distance matrix, are statistically significant. Feature abundance was normalized by relative abundance within each sample using the SILVA classifier (release 138). Graphs were generated using R (v.3.5.2). Differentially abundant taxa were identified using LEfSe with default parameters. Microbial metabolic functions were analyzed with PICRUSt2 to infer functional profiles, which were then mapped against the KEGG database for pathway abundance values. Additional diagrams were also created using R (v.3.5.2).

### Immunofluorescence staining

The sections were sequentially immersed in a series of xylene and ethanol solutions for dewaxing, followed by antigen retrieval with citric acid (pH 6.0). A 3% hydrogen peroxide solution was prepared using water, and the sections were placed in this solution in an incubator under ambient temperature for 20 minutes. Blocking was conducted with 10% goat serum at 37°C for half an hour. The primary antibodies, IL-17A (1:200 dilution, PTG, catalog number 26163–1-AP) and HIF-1α (1:200 dilution, bioss, catalog number bs-0737R), were diluted in an antibody dilution buffer and allowed to incubate overnight at 4°C. Goat anti-rabbit IgG-CY3 conjugated with horseradish peroxidase (HRP) (1:300 dilution, Servicebio) was prepared in PBST and kept at 37°C for 1 h. 4′,6-Diamidino-2-phenylindole solution was utilized to stain cell nuclei. Representative images were obtained using fluorescence microscopy (Nikon Eclipse C1, Tokyo, Japan). The number of positive cells was evaluated using Image Pro Plus (v.6.0) software.

### Enzyme-linked immunosorbent assay

The ELISA kit (ELM-IL17-1, Raybio) was used to measure IL-17A levels in the mouse serum. The experimental procedures followed the guidelines provided by the manufacturer. Absorbance readings were taken with an ELISA reader, and a standard curve was generated based on these values.

### Cell culture and cell wound scratch assay

Human keratinocytes (hacat) were purchased from IMMOCELL (Xiamen, Fujian, China). The cells were categorized into eight groups. In the normal medium control group (NC group), cells were grown in Dulbecco’s modified Eagle’s medium/F12 medium enriched with 10% fetal bovine serum and 1% penicillin–streptomycin liquid. In the high glucose medium control group (HC group), an additional 25 mM of glucose was added to the medium. In the IL-17A group, 100 ng/mL exogenous IL-17A (CM018-20HP; Chamot Biotechnology Co, Ltd, China) was added. The NI group was exposed to normal glucose medium along with IL-17A, while the HI group was treated with high glucose medium in combination with IL-17A. In the 2-DG group, 100 ng/mL exogenous IL-17A and 40 mM glycolysis inhibitor 2-DG (CD4251-1g, Coolaber) were added. Likewise, it encompasses the ND group as well as the HD group. In the BAY87-2243 group, 100 ng/mL exogenous IL-17A and 10 µM HIF1α inhibitor BAY87-2243 (87–2243, MCE) were incorporated. As before, it is divided into the NB group and the HB group. Following a 24 h incubation period, the cells covered the six-well culture plate. Subsequently, a cell scratch assay was conducted, and the scratch patterns of the cells at 0 and 24 h were recorded. The scratch area was calculated using ImageJ. The percentage of the scratch area (%) was calculated as (*A*_0_ − *A*_24_) / *A*_0_ × 100%, where *A*_0_ and *A*_24_ indicate the area of the scratch at 0 and 24 h. The levels of pathway-related proteins were assessed using WB.

### Western blot

The efficient radioimmunoprecipitation assay lysing buffer for tissue and cell samples (with phenylmethylsulfonyl fluoride) (SL1020-100mL, Coolaber) was used to extract cell proteins. The lysate was subsequently subjected to centrifugation at 12,000 × *g* at 4°C for 20 minutes to isolate the total protein. The protein concentration was measured using the BCA protein assay kit (EC0001, SparkJade). The membranes were moved and allowed to incubate overnight at 4°C with the specified primary antibodies: mouse monoclonal AKT (1:25,000; 60203–2-Ig; Proteintech), mouse monoclonal p-AKT (1:5,000; 66444–1-Ig; Proteintech), mouse monoclonal mTOR (1:25,000; 66888–1-Ig; Proteintech), rabbit monoclonal p-mTOR (1:1,000; 5536T; CST), rabbit polyclonal antibody anti-HIF1α (1:1,000; D222477-0025; Sangon Biotech), rabbit polyclonal antibody anti-HK2 (1:25,000; 22029–1-AP; Proteintech), mouse monoclonal HIF-1 (1:5,000; 66730–1-Ig; Proteintech), mouse monoclonal HK2 (1:10,000; 66974–1-Ig; Proteintech), and β-actin (1:20,000; T0022; Affinity). Afterward, the membranes were treated with the appropriate secondary antibodies, including HRP-conjugated goat anti-rabbit antibody (1:1000; A0208; Beyotime) and HRP-conjugated goat anti-mouse antibody (1:10, 000; SA00001-1; Proteintech) at room temperature for 2 h. Visualization of the blots was performed using the LAS4000 chemiluminescence system from Fujifilm in Tokyo, Japan, and the gray values of the films were analyzed using IPP software.

### Statistical analysis

An analysis of the statistical data were carried out utilizing SPSS (v.20.0). Data are presented as the mean ± standard deviation. In the case of multiple comparisons, one-way analysis of variance was employed and subsequently analyzed using the least significant difference post hoc test. The non-parametric Kruskal–Wallis test was applied to evaluate the pole test performance, grip strength test results, histological scores, ZO-1 integrity scores, and cell counts, with Mann–Whitney *U* post hoc testing. Comparisons between the two groups were conducted using independent *t*-tests. Spearman correlation analysis was performed with R (v.3.5.1) to assess correlations across different experiments. The results were considered statistically significant if the *P* value is <0.05.

### Conclusions

This research revealed the effect of FMT on promoting the healing of diabetic wounds. The experimental results demonstrated that FMT can regulate the microbiota imbalance in T2DM mice, restore it to the structure of healthy mice, promote the expression of IL-17A, accelerate the development of granulation tissue and the generation of collagen fibers, enhance glycolysis through the IL-17A–mTOR–HIF1α signal axis, accelerate the migration of keratinocytes, and accelerate the recovery of diabetic wounds.

## Data Availability

The data that support the findings of this study are fully available from https://doi.org/10.5061/dryad.n2z34tn7c.
